# Real time imaging of intra-axonal calcium flux in an explant mouse model of axonal Guillain-Barré syndrome

**DOI:** 10.1016/j.expneurol.2022.114127

**Published:** 2022-05-29

**Authors:** Madeleine E. Cunningham, Rhona McGonigal, Jennifer A. Barrie, Denggao Yao, Hugh J. Willison

**Affiliations:** Institute of Infection, Immunity, and Inflammation, College of Medical, Veterinary and Life Sciences, University of Glasgow, Glasgow, UK

**Keywords:** Guillain-Barr’e syndrome, Calcium imaging, Axonal injury, Calpain, Neuromuscular junction

## Abstract

The acute motor axonal variant of Guillain-Barré syndrome is associated with the attack of motor axons by anti-ganglioside antibodies which activate complement on the axonal plasma membrane. Animal models have indirectly implicated complement pore-mediated calcium influx as a trigger of axonal damage, through the activation of the protease calpain. However, this calcium influx has never been imaged directly. Herein we describe a method to detect changes in intra-axonal calcium in an ex vivo mouse model of axonal Guillain-Barré syndrome and describe the influence of calcium on axonal injury and the effects of calpain inhibition on axonal outcome.

Using ex vivo nerve-muscle explants from *Thy1*-TNXXL mice which axonally express a genetically encoded calcium indicator, we studied the effect of the binding and activation of complement by an anti-GD1b ganglioside antibody which targets the motor axon. Using live multiphoton imaging, we found that a wave of calcium influx extends retrogradely from the motor nerve terminal as far back as the large bundles within the muscle explant. Despite terminal complement pores being detectable only at the motor nerve terminal and, to a lesser degree, the most distal node of Ranvier, disruption of axonal proteins occurred at more proximal sites implicating the intra- axonal calcium wave. Morphological analysis indicated two different types of calcium-induced changes: acutely, distal axons showed swelling and breakdown at sites where complement pores were present. Distally, in areas of raised calcium which lacked detectable complement pores, axons developed a spindly, vacuolated appearance suggestive of early signs of degeneration. All morphological changes were prevented with treatment with a calpain inhibitor.

This is the first investigation of axonal calcium dynamics in a mouse model of Guillain-Barré syndrome and demonstrates the proximal reach of calcium influx following an injury which is confined to the most distal parts of the motor axon. We also demonstrate that calpain inhibition remains a promising candidate for both acute and sub-acute consequences of calcium-induced calpain activation.

## Non-standard abbreviations and acronyms

GBS: Guillain-Barré syndrome
AMAN: acute motor axonal neuropathy
MAC: membrane attack complex
NFil: neurofilament
Ab: antibody
NHS: normal human serum
NMJ: neuromuscular junction
MNT: motor nerve terminal

## 1 Introduction

Guillain-Barré syndrome (GBS) is an autoimmune paralytic disorder caused by inflammatory injury to the peripheral nervous system. Sub-types of GBS include both axonal (acute motor axonal neuropathy, AMAN) and demyelinating (acute inflammatory demyelinating poly-neuropathy, AIDP) variants. The extent of the paralysis experienced by patients is highly variable, as is the prognosis. Some patients recover rapidly within weeks (Ho et al., 1997a; Ho et al., 1997b; Soysal et al., 2011) whereas around 20% are still unable to walk unaided after 6 months (Willison et al., 2016). Residual limitations, ranging from mild to severe, are considered to be related to irreversible axonal injury (Bernsen et al., 1999; Bersano et al., 2006; Rees et al., 1998).

A proportion of severely paralysed AMAN cases do exhibit a remarkably rapid recovery, attributed either to injury restricted to the distal motor nerve and nerve terminal that regenerate rapidly (Ho et al., 1997a) or to reversible axonal injury that does not result in axonal transection, occurring at the nodes of Ranvier (NoR) and temporarily impairing conduction (Kuwabara et al., 1998; Uncini et al., 2013). It is not understood why some patients recover rapidly from acute axonal conduction block whereas others go on to develop an axonal injury that results in axonal transection with axon degeneration, but it is generally considered that the latter is the principal reason for delayed or failed recovery in GBS cases.

One mechanism of acute injury in axonal GBS is the targeted attack on the axon by autoantibodies against gangliosides that are localised to extracellular leaflet of the axolemmal membrane (O’Hanlon et al., 2001). Autoantibody binding to accessible segments of the axolemmal membrane, notably in distal motor nerves and spinal roots lying outwith the blood nerve barrier, triggers complement activation culminating in the deposition of complement membrane attack complex (MAC) pores into the membrane (Halstead et al., 2004; McGonigal et al., 2010; Susuki et al., 2007). We have previously demonstrated that this results in a rapid injury to axonal structural proteins that is mediated by calpain activation (McGonigal et al., 2010; O’Hanlon et al., 2003).

Since calpain is a calcium-activated protease, there is a presumption that there is significant calcium influx into the axon from the extracellular milieu through transmembrane complement pores. This has been demonstrated in an in vitro model of Miller Fisher syndrome (MFS), a GBS variant primarily associated with anti-GQ1b ganglioside antibody (Rodella et al., 2016). Although calcium is heavily inferred to be involved in the acute injury seen in our in vivo and ex vivo models of GBS it has never been demonstrated in a peripheral nervous system replete with axons, NoR and the motor nerve terminal (MNT), nor do we know the dynamics of calcium movement within or along the axon.

Calcium has acute cytotoxic effects and may also function as a more complex regulator of axonal degeneration. The mechanisms and pathways by which axons in GBS are destined to either recover through local membrane repair or proceed to focal transection with Wallerian degeneration of the distal stump are unknown. Changes in calcium concentration are associated with axon degeneration both as a result of dysregulation (Staal et al., 2010) and as part of programmed mechanisms of axon removal, such as Wallerian degeneration (Vargas et al., 2015). The recent concept of axons with raised intra-axonal calcium being able to persist in a metastable state (Williams et al., 2014), leading either to spontaneous recovery or destruction, well reflects the dichotomy of outcomes seen in AMAN patients. It is therefore also of interest to us to determine whether the same phenomenon occurs in our mouse model of AMAN.

In this study we addressed these gaps in our understanding by investigating the role of calcium in driving axonal injury using an ex vivo mouse model of GBS, in particular through directly visualising changes in axonal calcium levels. We first sought to determine whether changes in calcium can be measured in our mouse models. Next, whether influx of calcium through MAC pores can be linked to axonal injury or survival and lastly whether intervention with a calpain inhibitor would protect axons from calcium-mediated injury.

## 2 Materials and methods

### 2.1 Mice

Male and female *Thy1*-TNXXL mice aged 6–12 weeks were used for calcium imaging experiments (Williams et al., 2014). These mice are on a C57 Bl/6 background and express the fluorescence resonance energy transfer (FRET)-based calcium indicator, TNXXL in their neurons (Mank et al., 2008). TNXXL is a modified protein based on chicken skeletal muscle Troponin C, with cyan fluorescent protein (CFP) and citrine cp174 fused on either end of the protein. Upon binding of calcium, this genetically encoded sensor changes conformation allowing for ratiometric measurement of calcium changes in the axons of these mice. For immunofluorescent staining, C57 Bl/6 mice were used. Studies were carried out in accordance with the U.K. Animals (Scientific Procedures) Act, 1986.

### 2.2 Study design and statistics

Sample size was estimated using an a priori power analysis from initial studies with G*Power software (v 3.1.9.2), using an effect size of 2.01, an alpha error probability of 0.05 and a power of 0.9. Immuno-fluorescent images were analysed blinded. All data are displayed as mean ± SEM and relevant statistical tests and mouse numbers used for each analysis are described in the figure legends. Statistics were performed using GraphPad Prism (version 6.07).

### 2.3 Anti-ganglioside antibodies and normal human serum

Monoclonal anti-GD1b ganglioside antibody MOG1 (subclass IgG3) was previously generated by immunisation of GD3s^-/-^ ganglioside knock out mice with GD1b-KLH liposomes. The properties of this antibody and its binding pattern to GD1b ganglioside have been described previously (Greenshields et al., 2009). Antibodies against GD1b are classically associated with sensory forms of GBS. However, antibodies against GD1b have also been demonstrated to bind strongly in mouse motor nerves (Gong et al., 2002). We have shown that this antibody binds primarily to, and fixes complement on, neuronal membranes in mouse motor nerves without binding or injuring glial membranes (Cunningham et al., 2020). We therefore found this to be an ideal antibody for directing injury to the motor axonal membrane in this model of axonal GBS injury, even though this may not directly correspond with human AMAN serology. Normal human serum as a source of complement was taken from a single donor and frozen in aliquots at -80 °C.

### 2.4 Ex vivo injury model

Triangularis sterni (TS) nerve-muscle explants were used to visualise the motor nerve axons. This muscle is ideal for visualising distal motor nerves; it is a thin, flat muscle, densely innervated with branches of the intercostal nerves with many of its NMJs lying parallel to the muscle layers (McArdle et al., 1981). The explants can be kept alive for many hours in physiological solution, allowing for live imaging (Kerschensteiner et al., 2008). *Thy1*-TNXXL TS explants were dissected (Kerschensteiner et al., 2008) and mounted in oxygenated Ringer’s solution (116 mM NaCl, 4.5 mM KCl, 23 mM NaHCO_3_, 1 mM NaH_2_PO_4_, 11 mM glucose, 1 mM MgCl_2_ and 2 mM CaCl_2_). Explants were incubated with monoclonal anti-GD1b antibody (Ab) (100 μg/ml) for 30 min at 4 °C, a previously established protocol for effective binding of this Ab at the motor nerve terminal (Cunningham et al., 2016). Following the initial incubation with Ab, tissue was washed 3× with room temperature Ringer’s solution and then moved to the microscope for baseline imaging. Once a baseline image was established, Ringer’s solution was replaced with 40% NHS in Ringer’s solution for 10 min to initiate the complement-mediated injury. After 10 min, tissue was washed 3× in fresh Ringer’s and images were taken immediately and every 10 min for 1 h. For control tissue, the NHS step was omitted. The 10-min incubation time with NHS was analysed in pilot experiments to confirm this time was sufficient to cause terminal complement pore deposition at the motor nerve terminal. A priori, exclusion criteria for Ab+NHS preparations were set. Those preparations which did not show rises in calcium were to be tested for successful MAC deposition and excluded if negative. No preparations needed to be excluded in this manner.

In calpain inhibition studies, the model was performed as above with either 100 μM of the calpain inhibitor AK295 (McGonigal et al., 2010) or an equivalent volume of DMSO vehicle control added for 10 min along with 40% NHS.

### 2.5 Live imaging

*Z*-stacks of explant regions with high numbers of MNTs were acquired using a Zeiss LSM7 MP system, using a 20×/1.0NA water-immersion objective lens and a tuneable titanium/sapphire solid-state 2-photon excitation source (Chameleon Ultra II; Coherent Laser Group), tuned to 840 nm. Light was first filtered through a 685-nm LP dichroic. For FRET imaging, an LSM binary GaAsP photodetector module was used with CFP/YFP filterset cube (BP 455–500/BP 525–570) with an LP510 dichroic mirror.

After NHS incubation, tissue was rapidly rinsed 3×with Ringer’s and images were taken between 11 and 12 min after NHS was initially added. To minimise imaging time and prevent photodamage to the tissue, images were taken with 1.2–1.3 μm z-slice and pixel size of 754 nm with 2× line averaging.

### 2.6 Image analysis and quantification

**Fig. 1:**
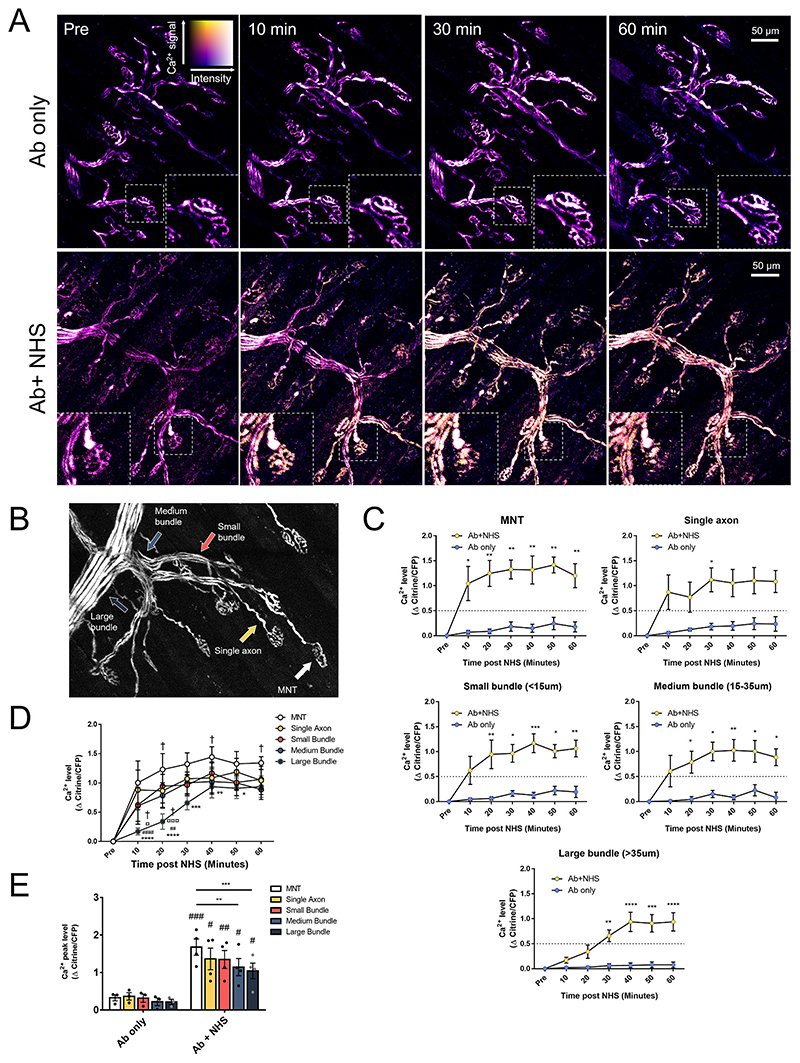
Axonal calcium influx after complement-mediated injury appears to occur as a retrograde wave starting at the MNT. A: Ratiometric calcium images from maximum intensity projections showing axonal calcium at different timepoints. Colour key demonstrates relative calcium levels, with “hotter” colours indicating higher calcium and brightness indicating fluorescent intensity. Insets show changes at individual MNTs. B: Image showing examples of axon categories: MNT, single axon, small (<15 μm), medium (15–35 μm) and large (>35 μm) bundles. C: Changes in calcium (Δ Citrine/CFP) over time (two-way repeated measures ANOVA with Sidak’s multiple comparisons test, * = *p* < 0.05, ** = *p* < 0.01, *** = *p* < 0.001, **** = *p* < 0.0001 vs Ab only). D: Graph combining Δ Citrine/CFP for axons of different types within Ab+NHS treated explants (two-way repeated measures ANOVA with Tukey’s multiple comparison’s test, * = vs MNT (*p* < 0.05), ** = vs MNT (*p* < 0.01), *** = vs MNT (*p* < 0.001), **** = vs MNT (*p* < 0.0001), ## = vs single axon (*p* < 0.01), #### = vs single axon (*p* < 0.0001), ¤ = vs small bundle (*p* < 0.05), ¤¤¤ = vs small bundle (*p* < 0.001) † = vs medium bundle (*p* < 0.05). E: Peak calcium levels of measured axons at different sites. For Ab only vs Ab+NHS, data were compared by a two-way repeated measures ANOVA with Sidak’s multiple comparison’s test). Within Ab+NHS treated explants; data were compared by a two-way repeated measures ANOVA with Tukey’s multiple comparison’s test). # = vs Ab only (*p* < 0.05), ## = vs Ab only (*p* < 0.01), ### = vs Ab only (*p* < 0.001), ** = *p* < 0.01, *** = *p* < 0.001. *n* = 3 Ab only, *n* = 4 Ab + NHS.

Ratios of citrine/CFP were calculated using the Fiji distribution of ImageJ software (version 1.52b) (Schindelin et al., 2012). Areas of interest were drawn overlying either a MNT, single axon (innervating an already measured MNT), or axons constituting a small bundle (< 15 μm), medium bundle (15–35 μm) or large bundle (>35 μm) as defined by (McGonigal et al., 2010)(Fig. 1B). For each of these areas, intensity measurements were taken of axonal citrine and CFP at each timepoint. Background measurements for each channel were also taken and subtracted from each measurement before calculating the ratio of citrine/CFP for each individual axon at each timepoint.

The morphology of axons was classed as either “unaffected”, “swollen” or “fragmented”. These measurements were only taken when the axon could be identified throughout the course of the experiment (some were not identifiable at some timepoints due to preparation drift, photobleaching or high background). Fragmented axons were those displaying separation between segments of the axon when the image stack was contrast adjusted.

Axons were classed as having “elevated” calcium if the change in citrine/CFP ratio exceeded 0.5 meaning a 50% rise in original axon calcium ratio as defined by (Williams et al., 2014). “Unchanged” axons remained below this threshold and axons scored as “returned” were previously above threshold before returning to below threshold values.

Calcium FRET images are displayed as ratiometric images. To create these, in the FIJI distribution of Image J, a maximum intensity projection (MIP) of *Z*-stack images was created, the CFP and citrine channels were split and a binary mask from one of these channels was created (whichever had the highest signal/noise ratio). Background was subtracted in each of the original channel MIPs before using the image calculator to multiply both the CFP and citrine MIPs by the binary mask. The ratiometric image was generated by dividing the resulting citrine x binary image by the CFP x binary image. Display was scaled to 8 bit (min set to 0 and max to 2) then a LUT (mpl-plasma) was applied to the resulting image. This image was then copied to Adobe photoshop (version CS5.1) along with the MIP of the single channel originally used as the binary mask. The ratiometric image was pasted as an overlay layer above the single channel image and despeckle filter was applied (Witte et al., 2019).

### 2.7 Immunofluorescence staining

To quantify presence of anti-GD1b antibody, complement and axonal proteins, immunofluorescence staining was performed. Explants from C57 Bl/6 mice were halved at the ribcage to allow 2 TS muscles from each animal, one to be used as Ab only control and one to be Ab+NHS treated. Injury was otherwise carried out as above (section: Ex vivo injury model). After treatment, explants were fixed in 4% paraformaldehyde, washed in PBS, 0.1 M glycine and PBS again for 5 min each. Tissue was then incubated in freezing ethanol (100%) for 10 min at -20 °C. Primary antibodies were added overnight in PBS with 3% (v/v) normal goat serum (NGS) and 0.5% (v/v) Triton X-100. Antibodies used were rat anti-MBP (Bio-Rad Cat# MCA409S, RRID:AB_325004; 1/500), mouse anti-phosphorylated neurofilament-H antibody SMI-31 (BioLegend Cat# 801602, RRID:AB_2715851; 1/1500), mouse anti-non-phosphorylated neurofilament-H antibody SMI-32 (BioLegend Cat# 801701, RRID:AB_ 2564642; 1/1500), mouse anti-MAC (Agilent Cat# M0777, RRID:AB_2067162; 1/50) and mouse anti-Ankyrin G (Thermo Fisher Scientific Cat# 33-8800, RRID:AB_2533145; 1/100).

Secondary antibodies were prepared at a 1/500 dilution in PBS with 3% NGS: Alexa Fluor 555 conjugated anti-rat IgG (T Thermo Fisher Scientific Cat# A-21434, RRID:AB_2535855), Alexa Fluor 646 conjugated anti-mouse IgG1 (Thermo Fisher Scientific Cat# A-21240, RRID: AB_2535809), Alexa Fluor 488 conjugated anti-mouse IgG2a (Thermo Fisher Scientific Cat# A-21131, RRID:AB_2535771) and Alexa Fluor 488 conjugated IgG3 (Thermo Fisher Scientific Cat# A-21151, RRID: AB_2535784). Quantitative images were taken on a Zeiss LSM 880 confocal microscope and representative images were taken on a Zeiss AxioImager Z1 microscope with ApoTome attachment.

### 2.8 FM-143 uptake

Ex vivo injury was performed as above (section: Ex vivo injury model), with the addition of Alexa Fluor 647-conjugated α-bungarotoxin (Thermo Fisher Scientific Cat# B35450; 1/500) alongside MOG1 antibody incubation. Instead of fixation, explants were loaded with FM 1–43 dye (Thermo Fisher Scientific Cat# T35356) at a final concentration of 2 μM in a high [K]^+^ Ringer’s solution (KCl concentration was increased to 56 mM and NaCl was reduced to 65 mM). Dye was loaded for 5 min at room temperature then explants were washed for 3 × 10 min in Ringer’s solution with no Ca^2+^, to reduce membrane activity and therefore destaining of FM 1–43. Explants were then viewed live on a Zeiss AxioImager Z1 microscope.

## 3 Results

### 3.1 Axonal calcium flux extends from the distal nerve in a retrograde manner

Axons in preparations which received only anti-GD1b Ab (Ab only) maintained baseline calcium levels throughout the imaging period (Fig. 1A,C). In explants which received anti-GD1b Ab and NHS (Ab+NHS), changes in calcium were seen as rapidly as 10 min after NHS addition. The most rapid and largest rise in calcium was induced in the distal part of the axon, the MNT at the NMJ. This reached significance shortly thereafter in the single axons innervating these MNTs and the axons of the small and medium bundles. Axons of large bundles showed a more gradual rise in calcium levels, remaining significantly lower than axonal calcium in single axons and axons of small and medium bundles until 30 min post-NHS and MNT calcium up to 50 min post-NHS (Fig. 1C, D). The calcium appeared, therefore, to behave like a retrograde wave which dissipated as it travelled further proximally from the MNT along the axon. This is further seen when measuring the average peak axonal calcium over the 60-min time course (Fig. 1E). While all NHS-treated axons are significantly higher than their Ab only treated regional-counterparts, peak calcium appears to trend downwards from the MNT to the large bundles and is significantly higher at the MNT than the peak calcium in medium and large bundles.

### 3.2 Axons with raised calcium are more likely to develop adverse changes in morphology

**Fig. 2:**
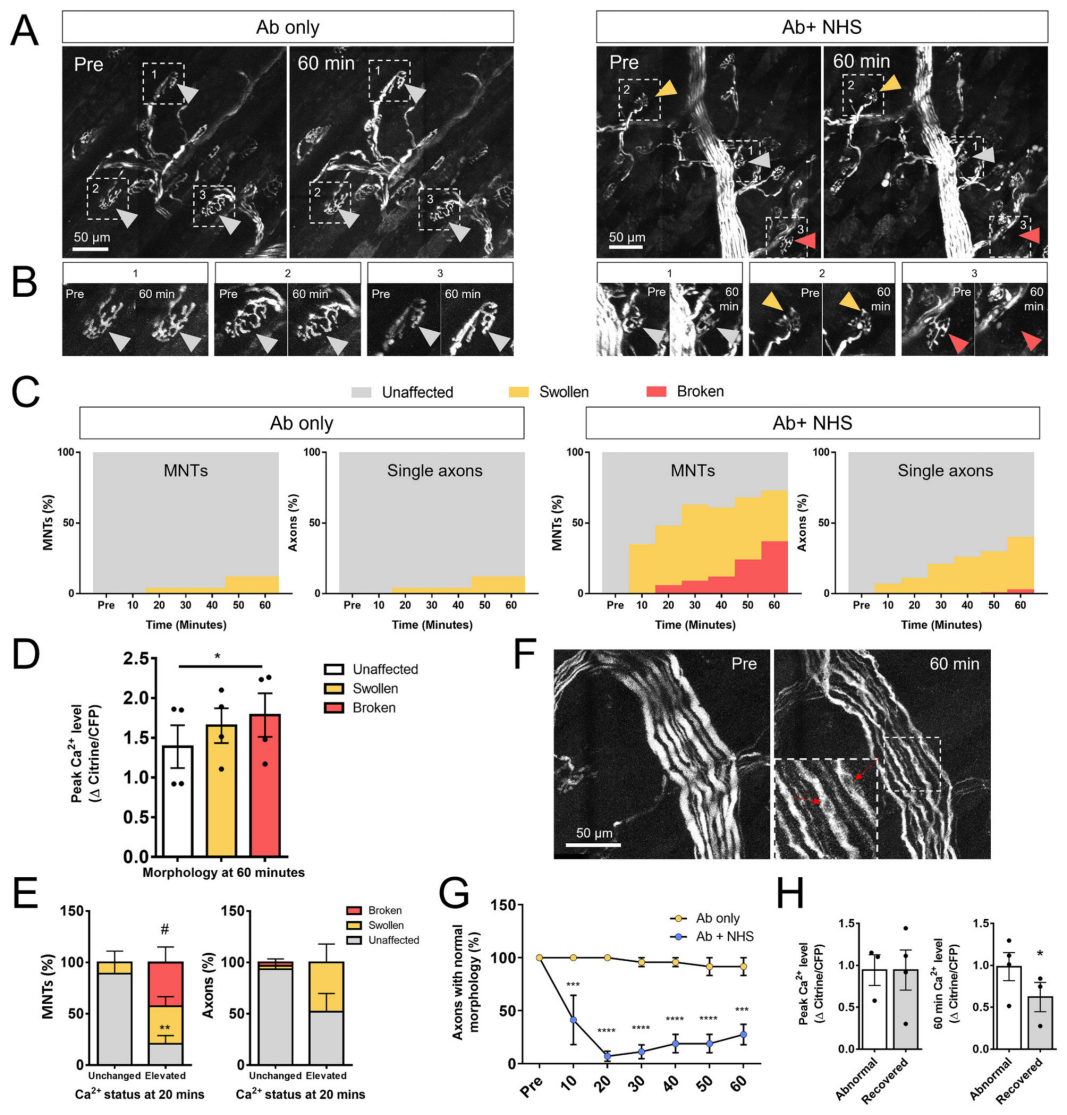
Calcium is associated with adverse morphological changes. A: Maximum intensity projections of single channel Z stacks showing morphological changes in axons which received either Ab only or Ab+NHS. Grey arrows show examples of axons which were unaffected, orange arrows show an example of a swollen MNT and red arrows show an example of a fragmented MNT. B: Close up of examples from A. C: Quantification of percentages of unaffected, swollen and fragmented axons at the MNT and single axons in Ab only and Ab+NHS treated explants over time. D: Peak calcium levels (max Δ Citrine/CFP) in axons which were either unaffected, swollen or fragmented at the 60 min timepoint. Fragmented axons had a higher peak calcium level than unaffected axons (one-way repeated measures ANOVA with Tukey’s multiple comparisons test, * = *p* < 0.05). E: Percentages of MNTs and single axons showing unaffected, swollen or fragmented morphology at 60 min after having either unchanged or elevated calcium at an early (20 min) timepoint. MNTs which had early elevation in calcium showed a higher percentage of fragmented morphology and a lower percentage were unaffected compared with axons which did not have an early rise in calcium (two-way ANOVA with Sidak’s multiple comparisons test - unchanged vs elevated: ** = unaffected, *p* < 0.01, # = fragmented, *p* < 0.05). F: Maximum intensity projection images showing changes in large bundle morphology. Axons appear spindly and wavy at 60 min post NHS. Red arrows in inset indicate examples of vacuoles. G: Percentage of large bundle axons showing normal morphology over the 60-min imaging period. Normal axons were significantly reduced compared to Ab only treated axons as soon as 10 min after NHS addition (two-way repeated measures ANOVA with Sidak’s multiple comparisons test, *** = *p* < 0.001, **** = *p* < 0.0001). H: Among Ab+NHS treated explants, peak axonal calcium (max Δ Citrine/CFP) between large bundle axons which either remained abnormal or had recovered by 60 min was no different; however, axonal calcium measured at 60 min was significantly reduced in large bundle axons whose morphology had recovered (paired *t*-test, * = *p* < 0.05). (For interpretation of the references to colour in this figure legend, the reader is referred to the web version of this article.)

Ab only treated explants showed no changes in axonal calcium levels and the morphology remained stable throughout the imaging process, only developing a small number of swollen axons (12% by 60 min for both MNT and single axons, Fig. 2A,C). In Ab+NHS treated explants, 36% of MNT were swollen and 37% were severely disrupted (referred to as fragmented) by 60 min. Single axons’ morphological changes were less severe with 37% swollen by 60 min but only 3% fragmented (Fig. 2A,C). Calcium levels at the MNT were associated with axonal fate: MNT axons which went on to fragment had a higher peak calcium level than those which remained unaffected by the 60-min time point. Axons which remained swollen by the end of imaging had calcium levels somewhere between unaffected and fragmented axons (Fig. 2D). Early changes in calcium can predict the fate of distal axons: in particular, axons at the MNT whose calcium was elevated by 20 min showed a higher presence of “fragmented” morphology by 60 min than those axons which did not see an early rise in calcium (Fig. 2E). Similarly, the single axons innervating these MNTs saw a higher presence of “swollen” morphology after early elevations in calcium, but this did not reach significance. No “swollen” or “fragmented” axons were seen in small, medium or large bundles (data not shown), though other changes were noted, particularly in axons of large bundles. Axons of large bundles developed a spindly, undulating appearance with prominent cytoplasmic vacuolation (Fig. 2F). Similar phenotypes were sometimes seen in medium and small bundle axons but due to their smaller axon diameter it was less clear when qualitatively scoring these than for larger axons. Most large bundle axons showed a reduction in normal morphology by 20 min post NHS (notably, earlier than a detectable rise in calcium in these axons vs control, Fig. 1C), but some axons did begin to recover a normal morphology by the end of the imaging period (Fig. 1G). The peak calcium levels in axons which recovered by 60 min was not different from those which retained an abnormal morphology at this timepoint. However, the recovered axons did have significantly lower calcium levels at the 60-min timepoint (Fig. 2H), implying that a recovery in calcium correlates with a recovery in axon morphology. Further investigation of these vacuole-like structures found that they were not associated with markers for mitochondria, lysosomes or endosomes (data not shown). We therefore hypothesise that they are most likely axonal vacuoles, akin to those observed in Wallerian degeneration of central nervous system axons following optic nerve transection (Beirowski et al., 2010).

### 3.3 Retrograde intra-axonal spread of calcium may cause axonal injury at sites lacking direct antibody and complement pore deposition

**Fig. 3:**
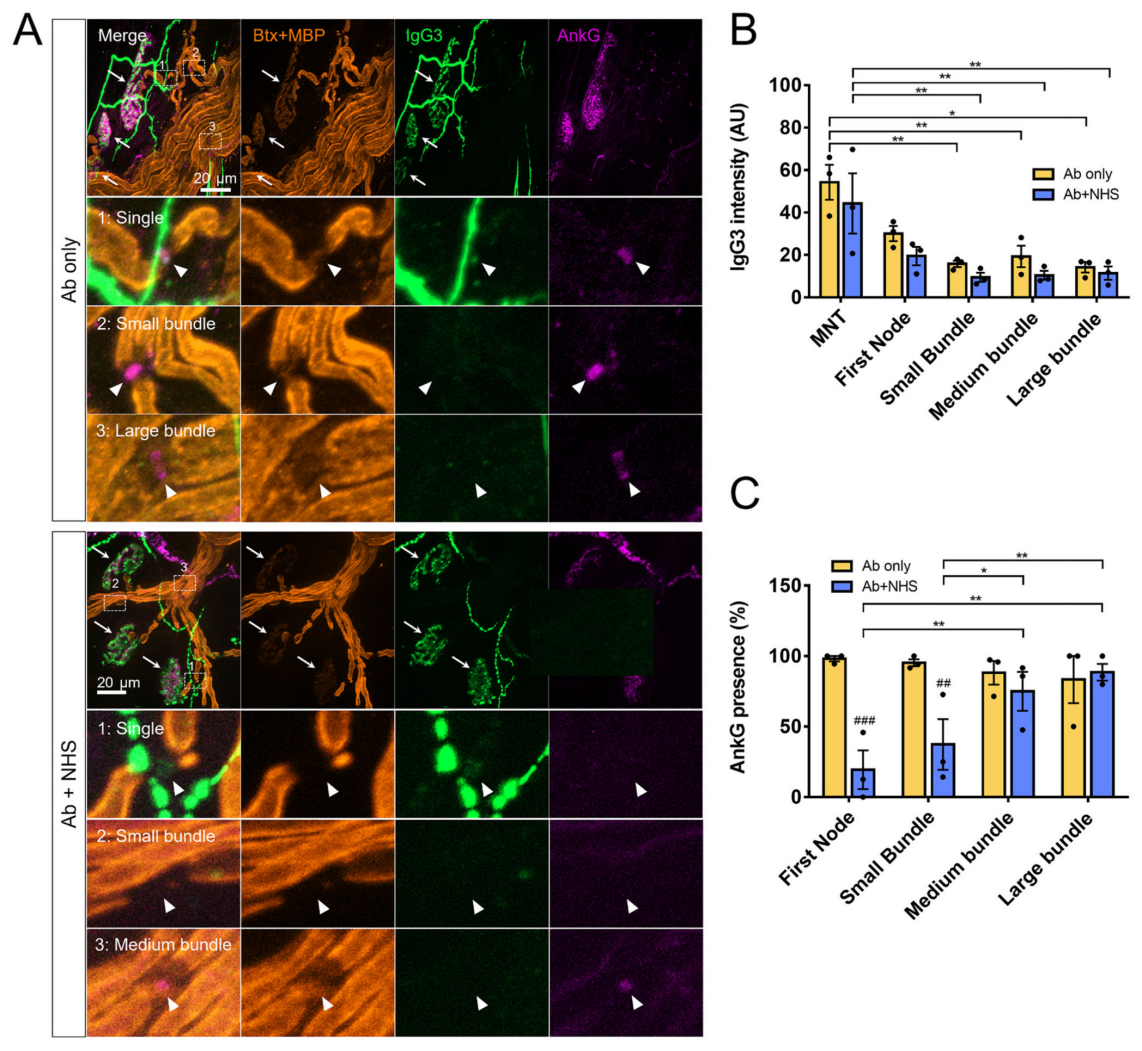
Fluorescent staining for anti-GD1b IgG3 antibody and AnkG in the distal nerve. A: Illustrative images of anti-GD1b IgG3 antibody deposition and nodal protein AnkG. In Ab only treated explants deposition of Ab can be seen overlying the MNT (shown by white arrows) and first node of Ranvier (Ab only, inset 1). AnkG is present at single axon nodes, and nodes of small and medium bundles. In Ab+NHS treated explants, deposition of IgG3 is similarly seen at MNT and first node. AnkG is not present at first nodes or nodes or small bundles (Ab+NHS, inset 1,2). However, AnkG is present at more proximal sites (Ab+NHS, inset 3). B: Quantification of IgG3 staining intensity. C: Quantification of AnkG presence at nodes. ## = vs Ab only (*p* < 0.01), ### = vs Ab only (*p* < 0.001), two-way repeated measures ANOVA with Sidak’s multiple comparison’s test. * = *p* < 0.05, ** = *p* < 0.01, two-way repeated measure ANOVA with Tukey’s multiple comparisons test. n = 3 per group.

**Fig. 4:**
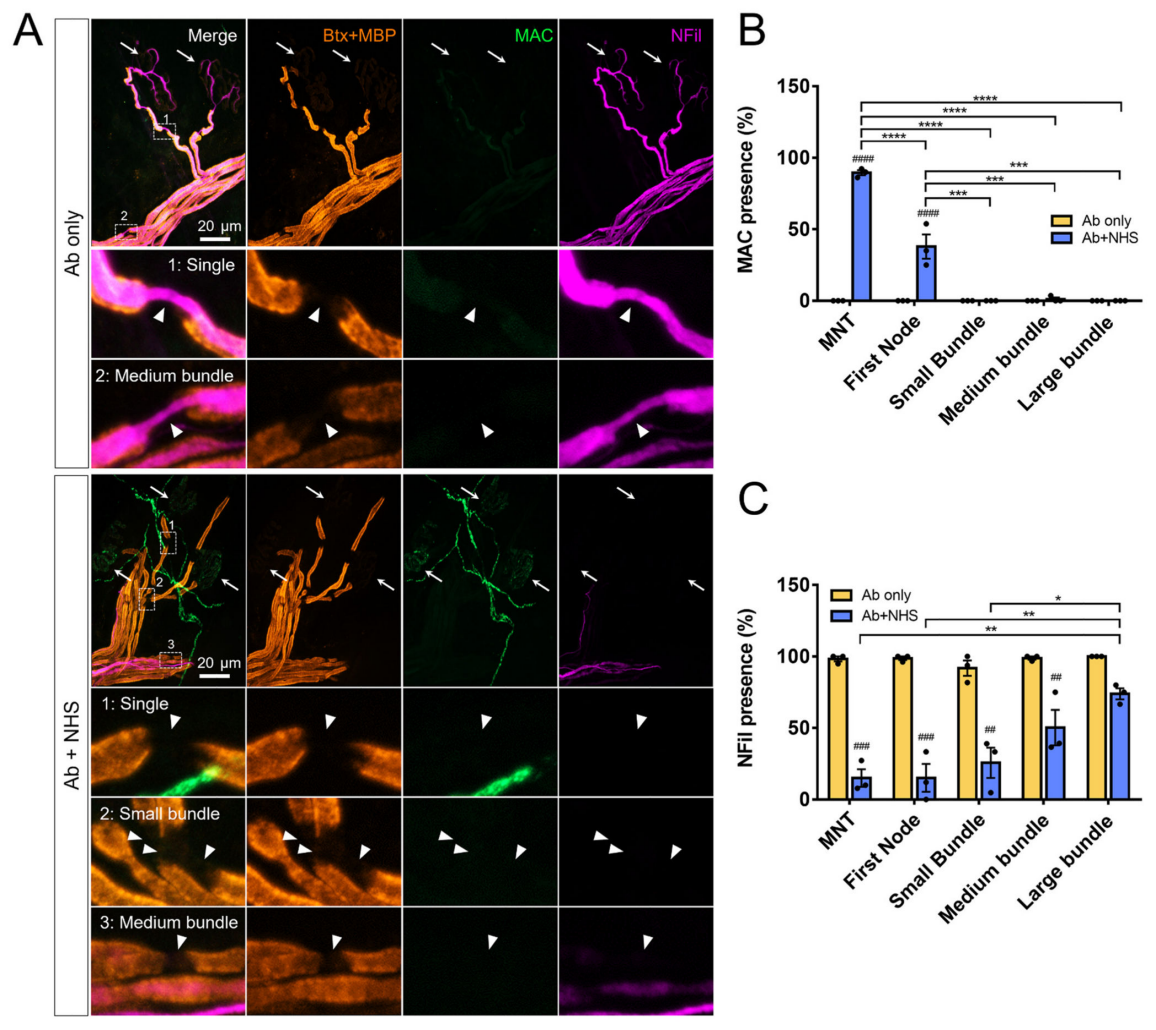
Disruption to NFil extends further proximally than deposition of MAC. A: Illustrative images of MAC deposition and axonal protein NFil. No MAC deposition was seen at the distal nerve in Ab only treated explants. NFil was present all along the distal axon, including the MNT (white arrows indicate MNT, insets show NFil at single axon and medium bundle nodes). In Ab+NHS treated explants; MAC deposition is observed at the MNT. The MNT, single axons, small bundle axons and some axons of medium bundles show a loss of NFil staining (Ab+NHS inset 1,2,3). B: Quantification of MAC presence. C: Quantification of NFil intensity. ## = vs Ab only (*p* < 0.01), ### = vs Ab only (*p* < 0.001), #### = vs Ab only (*p* < 0.0001), two-way repeated measures ANOVA with Sidak’s multiple comparison’s test. * = *p* < 0.05, ** = *p* < 0.01, *** = *p* < 0.001, **** = *p* < 0.0001, two-way repeated measure ANOVA with Tukey’s multiple comparisons test. n = 3 per group.

Deposition of anti-GD1b antibody (as judged by IgG3 immunostaining) was found to be primarily at the MNT and first NoR proximal to the MNT. Deposition of IgG3 was not seen at nodes of small, medium and large bundles. As both controls and injured explants received anti-GD1b antibody, this pattern of staining was seen in both Ab only and Ab+NHS treated groups (Fig. 3A,B). Deposition of the terminal complement component, membrane attack complex (MAC), was not seen in Ab only treated explants. In Ab+NHS treated explants, significant presence of MAC was seen at the MNT and at the first NoR when compared to Ab only control, though this was significantly lower at the first NoR compared to MNT (around 90% of MNT compared with 38% at first NoR, illustrative image shows example of first NoR lacking MAC deposit). Presence of MAC was not seen at nodes of small medium and large bundles (Fig. 4A,B). Axonal proteins neurofilament heavy (NFil) and ankyrin G (AnkG), both of which are calpain substrates, were used as a measure of axonal injury, indirectly demonstrating Ca^2+^ presence and calpain activation. Presence of AnkG at the first node and nodes of small bundles was significantly reduced in Ab+NHS treated explants compared with Ab only treated controls. Within the Ab+NHS treated group, presence of AnkG was also significantly lower at the first node and nodes of small bundles when compared to nodes of both medium and large bundles (Fig. 3A,C). The presence of NFil staining was reduced in Ab+NHS treated explants vs Ab only at all measured sites except large bundles. Within Ab+NHS treated explants, NFil intensity was significantly lower at the MNT, first node and nodes of small bundles compared to the nodes of large bundles (Fig. 4A,C). Therefore, despite IgG3 anti-GD1b antibody and MAC being only significantly detectable at the MNT and the first node of Ranvier, nodal AnkG and NFil are disturbed as far proximally as the small and medium bundles, respectively.

### 3.4 Calpain inhibition prevents morphological changes at the distal nerves

**Fig. 5:**
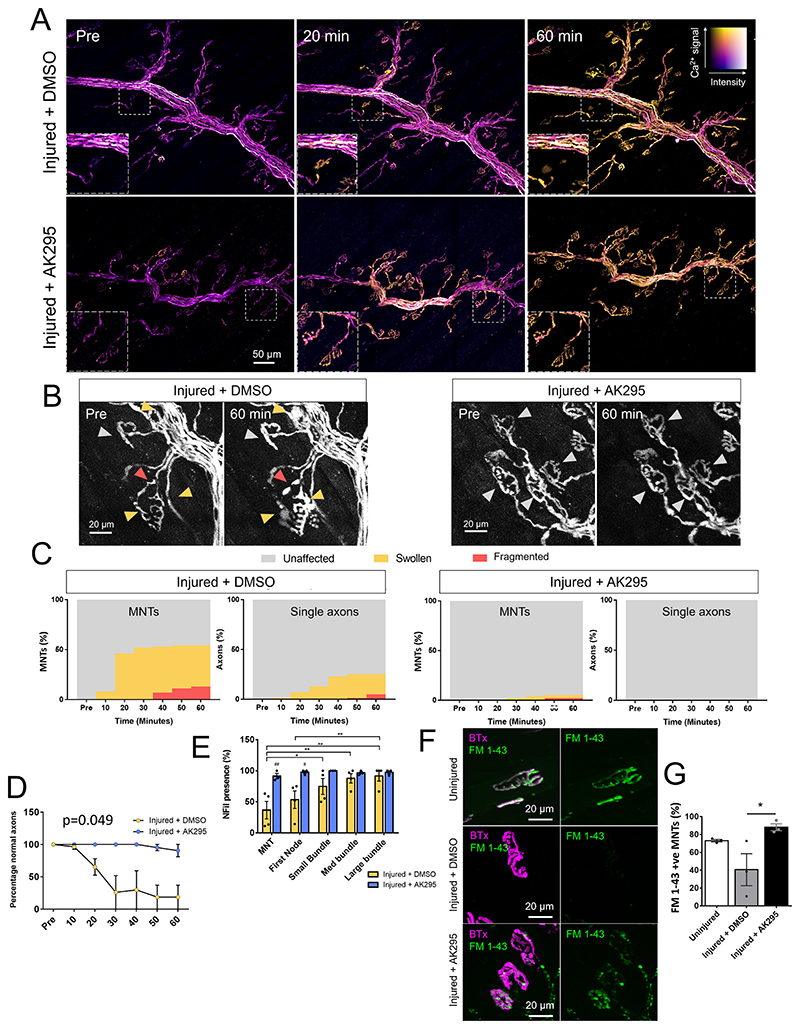
Calpain inhibition prevents adverse morphological changes in the axon without affecting calcium influx. A: Ratiometric calcium images from maximum intensity projections showing axonal calcium at different timepoints. Colour key demonstrates relative calcium levels, with “hotter” colours indicating higher calcium and brightness indicating fluorescent intensity. Insets show changes at individual MNTs. B: Maximum intensity projections of single channel showing morphological changes at MNT in explants which were injured using Ab+NHS with the addition of either AK295 or DMSO vehicle control. Grey arrows = unaffected axons, yellow arrows = swollen axons and red = fragmented axons. C: Quantification of percentages of unaffected, swollen and fragmented axons at the MNT and single axons in DMSO or AK295 treated injured explants. D: Morphological changes seen in large bundles (*p* = 0.049 two-way repeated measures ANOVA with Sidak’s multiple comparisons test). n = 4 Injured + DMSO, n = 3 Injured + AK295 E: Quantification of NFil (top) and AnkG (bottom) presence. # = *p* < 0.05, ## = vs Ab only (*p* < 0.01), two-way repeated measures ANOVA with Sidak’s multiple comparison’s test. * = *p* < 0.05, ** = *p* < 0.01, *** = *p* < 0.001, two-way repeated measure ANOVA with Tukey’s multiple comparisons test. *n* = 4 Injured+DMSO, *n* = 3 Injured+AK295 F: Illustrative images showing uptake of FM 1–43 at the MNTs of uninjured, injured + DMSO or injured + AK295 explants. Staining in both injured groups was more punctate than uninjured explants. G: Quantification of FM 1–43 presence at MNTs (*p* < 0.05, one-way ANOVA with Tukey’s multiple comparisons test). *n* = 3 for both groups. (For interpretation of the references to colour in this figure legend, the reader is referred to the web version of this article.)

Calcium influx was unaltered in the presence of the calpain inhibitor AK295: there was no change from control injured group (injury + DMSO vehicle) when explants were treated with AK295 (Bartus et al., 1994), an inhibitor of calpains I and II (Fig. 5A, Supplementary 1A). However, morphological changes seen in the control injured group, such as swelling (41% at 60 min) and breaking (13% at 60 min) of the MNTs were not seen in AK295-treated explants. Only 3% of MNTs were swollen and 2% fragmented at 60 min in the AK295-treated group (Fig. 5B, C, Supplementary 1B). Single axons were less affected than axons at the MNT in control injured groups with 36% of single axons showing swollen morphology and 4% fragmented. This was again improved in the AK295-treated group which showed no swollen or fragmented single axons. No swollen or fragmented axons were seen in the axons of small, medium and large bundles in either group (data not shown). Morphological changes which were previously seen in the large bundles of Ab+NHS treated explants were then investigated in injured explants which were treated with either DMSO or AK295. AK295 treated preparations showed significantly fewer abnormal axon morphologies in the large bundles compared with DMSO-treated control (Fig. 5D), indicating a rescue of this phenotype with inhibition of calpain. To confirm that the loss of NFil and AnkG presence was due to the calcium wave activating calpain at sites proximal to MAC pore deposition, explants from injured + DMSO and injured + AK295 groups were stained for NFil and AnkG. The reduction in NFil seen at the MNT and first NoR in Injured + DMSO treated explants was significantly protected in injured + AK295 treated explants. Similarly, the reduction of AnkG seen at the first NoR was also significantly protected in injured + AK295 treated explants (Fig. 5E). To confirm that loss of Nfil staining using SMI-31 antibody, which detects phosphorylated Nfil-heavy was not due to loss of phosphorylation of the protein, we also performed immunofluorescence staining with SMI-32, which detects non-phosphorylated NFil-heavy. We found a similar loss of staining using SMI-32 antibody (Supplementary 1C) in the injured + DMSO group. This, plus the protection of Nfil staining presence using both antibodies in AK295-treated explants suggest these changes are indeed calpain related.

To investigate the mechanism through which AK295 might prevent swelling of the axon, particularly at the MNT, we compared the ability of nerve terminals to uptake FM 1–43. FM 1–43 is a lipophilic styryl dye used as a plasma membrane probe and labels vesicle pools by being endocytosed at the MNT. This tests whether the machinery involved in vesicle retrieval by endocytosis was being affected by calpain-mediated injury and, therefore, if swelling was in part due to a failure in this process. If this was the case, AK295 treatment may be preventing swelling at the MNT by allowing reuptake of vesicles into the axon terminal by endocytosis. AK295 treated explants demonstrated a greater ability to uptake FM 1–43 at their nerve terminals than control injured (injured + DMSO vehicle) explants (Fig. 5F,G). The DMSO treated group had a higher variation between explants. Staining in AK295 treated and, where present, in control injured MNTs was more punctate than in completely uninjured explants, indicating calpain inhibition does not completely prevent complement-mediated changes within the synapse (Fig. 5G).

## 4 Discussion

This study demonstrates that changes in axonal calcium can be readily visualised in the distal motor nerves of a mouse model of GBS. The targeted nature of the anti GD1b Ab directed injury induced in this ex vivo model restricts the deposition of complement MAC pores to the MNT and to a lesser degree the most distal node of Ranvier (first node). Despite this, changes in calcium were observed to extend proximally up motor axons, even as far as large nerve bundles. We consider that this retrograde wave of calcium is attributable solely to calcium influx through MAC pores at the MNT and the first node that then travel retrogradely along the axon, rather than through complement pores that may have directly deposited on the nodal axolemma locally in large bundles themselves. However, the wave of calcium may be supplemented by other sources of calcium. Calpains and other proteases have previously been demonstrated to cleave voltage gated sodium channel α-subunits (Bano et al., 2005; von Reyn et al., 2012), the disruption of which causes abnormal Na^+^ influx, resulting in dysregulation of intra-axonal calcium by both reversal of the sodium-calcium exchanger and opening of voltage gated Ca^2+^ channels (Wolf et al., 2001). These disruptions can cause prolonged increases in intra-axonal calcium. Calpain inhibition did not affect the retrograde wave of calcium in this instance, however activation of other proteases cannot be ruled out. Other potential sources of calcium which may propagate the wave may be from intra-axonal sources such as disrupted mitochondria (Civera-Tregon et al., 2021; Mironov et al., 2005) – an occurrence which has previously been demonstrated at the MNTs in GBS mouse models (Halstead et al., 2004), or from the endoplasmic reticulum (Villegas et al., 2014). Further investigation of the possible sources of intra-axonal calcium that might contribute to the intra-axonal calcium excess is warranted.

Regardless of the source of the retrograde calcium wave we observed, it appears to have major consequences for axon health. Live morphological analysis demonstrated that injured preparations showed a higher propensity for adverse morphological changes at the MNT and single axon and that this was significantly correlated with higher axonal calcium. We concluded that this is due to direct influx of calcium at the MNT/first node, as immunofluorescent analysis revealed these were the only anatomical areas at which antibody and complement MAC pore were detectable. It remains possible that complement pores were deposited at more proximal sites at a threshold below detection by immunofluorescence staining. Immunofluorescent analysis of axonal proteins demonstrated that despite antibody and MAC pore being only detected at the MNT and first node, the integrity of the axonal proteins AnkG and NFil is disturbed retrogradely as far back as small and medium bundles respectively. AnkG and NFil are both known calpain substrates (Schafer et al., 2009; Schlaepfer et al., 1985). This implies that the retrograde wave of calcium, despite diminishing in intensity as it travels proximally, is still able to activate calpain at contiguous intra-axonal sites with no direct complement deposition in the adjacent axolemmal membrane.

Retrograde calcium signalling has been demonstrated previously in zebrafish larvae and cultured mouse neurons following axotomy. Vargas et al. demonstrated an initial retrograde calcium wave that was later followed by a “terminal wave” which directly preceded axon fragmentation, that was prevented by expression of WLDs, a mutation which originally occurred spontaneously in mice which delays the onset of Wallerian degeneration (Vargas et al., 2015). In the current study, following the initial wave of intra-axonal calcium, we did not see a return to baseline calcium levels within this imaging period. This presumably occurs since MAC pores remain present and potent for ion flux throughout the imaging period, preventing any restoration of calcium homeostasis. The timeline in our studies follows that described by Williams et al., in which axonal mechanopores induced by contusion injury allow calcium to enter the axon and thereby cause calpain-mediated effects (Williams et al., 2014). The axonal injury in this latter model was WLDs independent, therefore not caused by programmed axon degeneration. We have previously investigated the effects of WLDs in a mouse model of MFS and found that it similarly did not affect the extent of axon degeneration initiated by deposition of MAC pores in the axon (Willison, 2005).

Using live imaging we were able to demonstrate morphological changes in the more proximally sited large bundles in response to antibody and complement-mediated injury, a previously unreported observation in our model. Large bundles (those which are >35 μm in diameter) began to display a spindly, undulating phenotype with the appearance of cytoplasmic vacuolation, a phenotype which was prevented by calpain inhibition. These axons did not have detectable deposition of MAC, nor changes in axonal proteins NFil and AnkG but did show raised calcium. It therefore remains possible that calpain was activated and that other axonal calpain substrates (reviewed in Ma, 2013) not investigated here could be disrupted causing changes in axonal structure. However, of particular note, significant changes in large bundle axon morphology occurred before significant calcium rise in these axons, implying that, in fact, activation of calpain at more distal sites is the trigger for these changes. This phenotype appears, therefore, to be distinct from the more acute changes happening at the MNT and single axons and may be indicative of a precursor to axon degeneration. Peak calcium levels did not differ between large bundle axons which remained abnormal or regained their normal phenotype. This is similar in concept to the calcium metastable state, where axons may have similarly raised calcium levels, but may either return to normal or proceed to break down. During this imaging period we did not witness axons of large bundles undergoing breakdown so it is unknown whether this would have been the ultimate consequence for those axons whose morphology remained abnormal. A similar axonal phenotype was identified preceding secondary axon degeneration following demyelination (El Waly et al., 2020) in peripheral nerve and another study noted formation of vacuoles in the distal stump of lesioned optic nerves (Beirowski et al., 2010). In the latter study, mitochondria were also confirmed not to cluster or coincide with swellings containing these vacuoles. These phenotypical changes may therefore be an early indicator of axonal degeneration, particularly since the phenotype was prevented with inhibition of calpain, a known mediator of axonal degeneration (Ma et al., 2013). This prevention by calpain inhibition also implies that the observed phenotype was specifically calpain related, and not due to other consequences of raised calcium in the axon, nor of any other effects of NHS addition into the explant preparations. As stated, WLDs does not prevent acute axon injury in our mouse models but it would be of interest to see whether the morphological changes observed in large bundles could be prevented in WLDs mice, as this would further imply these changes in large bundle axons were early signs of degeneration. As noted, changes were also seen in axons of small and medium bundles, but these were often less obvious due to the smaller axon diameter and therefore less easily quantified.

Calpain inhibition was also shown to prevent the acute effects of antibody and complement deposition at the distal nerve sites (MNT and single axon). We have shown previously that inhibition of calpain prevents damage to structural proteins at these sites, without preventing loss of axonal function due to the deposition of MAC pores and influx of extracellular ions (McGonigal et al., 2010; O’Hanlon et al., 2003). We therefore expected calpain inhibition to prevent breakdown of the distal axon, without preventing swelling. This hypothesis is consistent with previous studies in which calpain inhibition prevented breakdown but not swelling of CNS axons after spinal cord injury (Williams et al., 2014) and in which calpain inhibition did not prevent formation of axolemmal bulges in cultured neurons treated with snake toxins (Duregotti et al., 2013). Therefore, calpain was not responsible for the membrane rearrangement which caused these swellings. However, our observations did show that inhibition with AK295 prevented not only breakdown of axons but also swelling, implying calpain is responsible for membrane reorganisation, in particular at the MNT and distal axon. Since the MNT is a very specialised site, we consider that calpain may have a different role in comparison with that in cultured neurons or intact spinal cord axons. In some contexts, swelling at the MNT is due to an imbalance in synaptic *exo*- and endocytosis in which vesicle membranes cannot be retrieved, therefore increasing the surface area of the MNT membrane. Calpains do have physiological roles in synaptic transmission (reviewed in Wu and Lynch, 2006) but inappropriate activation of calpain has also been shown to prevent endocytosis (Rudinskiy et al., 2009; Wu et al., 2007). Our data suggest that in our model, this latter effect may be relevant as injured membranes with calcium influx and unwanted calpain activation did not have efficient vesicle retrieval, as measured by reduced uptake of FM 1–43. This was protected by calpain inhibition, indicating the ability to retrieve vesicles from the membrane may rescue the swollen appearance of MNTs. Although the single axons were not as severely affected by swellings as the MNT, their change in morphology may be as a downstream consequence of swelling at the MNT.

We have used an ex vivo nerve-muscle preparation to live image these changes in calcium. As we wanted to demonstrate what calcium changes occur following anti GD1b Ab and complement-mediated injury to the motor nerve terminal and distal motor axon, we have used a highly reliable and reproducible monoclonal antibody against GD1b ganglioside. Whilst GD1b is commonly associated with predominantly sensory forms of human GBS, we have found in this ex vivo mouse model, that the anti-GD1b antibody very specifically and solely binds to the target ganglioside on the motor axonal membrane. More clinically relevant antibodies, such as the anti-GM1 monoclonal antibody DG2 we have used extensively (Greenshields et al., 2009) have the caveat that they also bind to glial membranes and resident kranocytes at the NMJ, and therefore do not induce a purely motor axonal injury (McGonigal et al., 2022; Court et al., 2008). Antibodies to another clinically relevant ganglioside, GD1a, lack the ability to consistently cause injury in wild type mice, requiring the overexpression of ganglioside as seen in GD3s^-/-^ (Goodfellow et al., 2005). The GD1b antibody chosen for this study has been extensively used in our previous in vivo and ex vivo models of GBS to injure nerve terminals and distal nerve without concomitantly binding glial membranes (Cunningham et al., 2020). It was therefore deemed a suitable candidate for inducing motor axonal toxicity in this ex vivo experimental model setting. This may be a reflection of subtle differences in human and mouse ganglioside composition or concentration in motor nerves; however, in in vivo models the anti-GD1b mAb does also bind to sensory motor nerve components including the DRG, and this could therefore complicate the translatability of these ex vivo findings into in vivo settings (Cunningham et al., 2016).

## 5 Conclusions

This study uses live calcium imaging to investigate changes in axonal calcium following autoimmune injury to the peripheral nerve motor axon. It substantiates the missing link between the known deposition of complement pores in the membrane and the downstream consequences of calpain-mediated axonal injury. Here, we have used calcium imaging to reveal the extent of calcium influx resulting from deposition of autoantibody and complement at only very distal sites adjacent to nerve terminals, and also observed that damage may extend further than localised areas of complement deposition. Our data are also consistent with the concept of a calcium-dependent metastable state of axonal viability, in which elevated intra-axonal calcium does not necessarily imply an axon is destined for Wallerian-like degeneration but could potentially undergo local repair. These data highlight a therapeutic window of opportunity in which calpain inhibition could prove to be a promising candidate pathway for clinical studies in the motor axonal injury occurring in GBS.

## Supplementary Material

Supplementary 1 Supplementary data to this article can be found online at https://doi.org/10.1016/j.expneurol.2022.114127.
