# Supplementary material for: Real time imaging of intra-axonal calcium flux in an explant mouse model of axonal Guillain-Barré syndrome
Source: Exp Neurol. Author manuscript; Available in PMC 2023 Feb 21. (PMC7614209; doi:10.1016/j.expneurol.2022.114127)
Supplement: Supplementary 1 — Supplementary data to this article can be found online at https://doi.org/10.1016/j.expneurol.2022.114127. [file EMS164618-supplement-Supplementary_1.pdf]

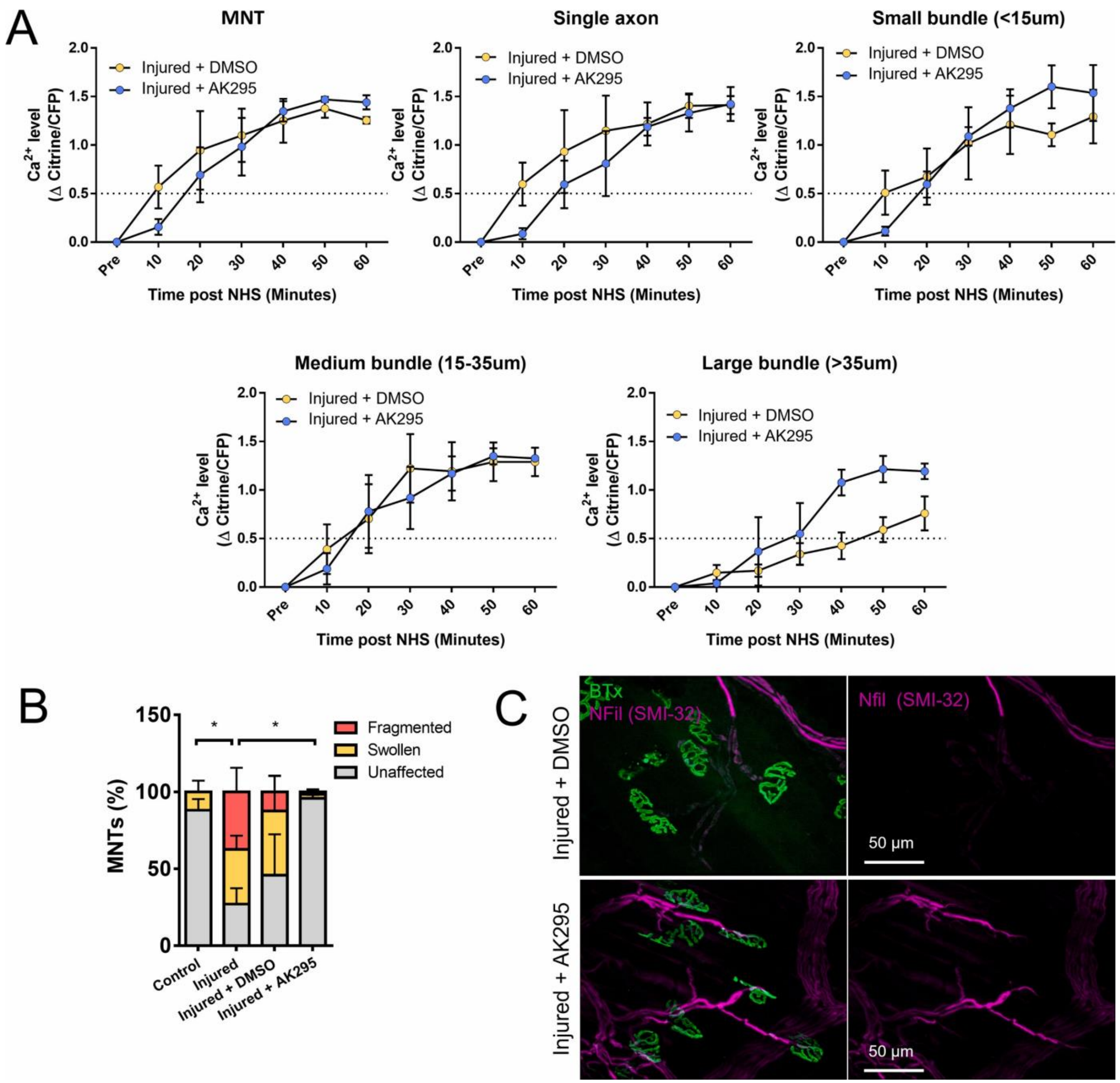

Supplementary 1. A: Changes in calcium (Δ Citrine/CFP) over time in explants which received injury with Ab+NHS with the addition of either AK295 or DMSO vehicle control. No significant differences were observed between groups (two-way repeated measures ANOVA with Sidak's multiple comparisons test). B: Morphological changes at the MNTs in each experimental group at 60 min. * = unaffected ($p < 0.05$), two-way ANOVA with Tukey's multiple comparisons test. $n = 3$ control, $n = 4$ Injury, $n = 4$ Injured+DMSO, $n = 3$ Injured+AK295. C. Illustrative images showing SMI-32 immunostaining for non-phosphorylated Nfil-H. A similar loss of staining is seen as with antibody for phosphorylated Nfil-H, in injured preparations with DMSO vehicle. In AK295-treated explants, SMI32 staining was protected.
